# The Association between Social Support and Cognitive Impairment among the Urban Elderly in Jinan, China

**DOI:** 10.3390/healthcare9111443

**Published:** 2021-10-26

**Authors:** Feng Jiang, Fanlei Kong, Shixue Li

**Affiliations:** 1 Centre for Health Management and Policy Research, School of Public Health, Cheeloo College of Medicine, Shandong University, Jinan 250012, China; whoops@mail.sdu.edu.cn; 2 NHC Key Lab of Health Economics and Policy Research, Shandong University, Jinan 250012, China

**Keywords:** social support, cognitive impairment, elderly, urban, China

## Abstract

China is currently facing a severe challenge of population ageing. However, no study has specifically explored the association between social support and cognitive impairment in Chinese urban elderly aged 60 and older. We explored the prevalence of cognitive impairment and its relationship with social support among the urban elderly aged above 60 years in Jinan, China. A total of 522 urban elderly individuals were recruited using multi-stage cluster random sampling, of which 35.55% were males and 64.45% were females. The average age of all participants was 69.66 ± 8.91 years old. Social support was assessed using the Social Support Rating Scale. Cognitive status was assessed using the Mini-Mental State Examination. Data were collected through face-to-face interviews using structured questionnaires. Descriptive analysis, chi-square tests, and logistic regression analyses were conducted. After analysing the data from 512 participants, 154 (30.1%), 352 (68.8%), and 6 (1.2%) participants had high, moderate, and low levels of social support, respectively. In addition, 125 participants (24.4%) had a cognitive impairment, while the other 387 participants (75.6%) had a normal cognitive status. Binary logistic regression analyses showed that age, educational level, occupation or pre-retirement occupation, and social support were statistically associated with cognitive impairment. A significant association between social support and cognitive impairment was found among the urban elderly in Jinan, China, which provided useful information for the intervention of cognitive impairment. More attention should be paid to the social support of the urban elderly to effectively reduce the occurrence of cognitive impairment.

## 1. Introduction

China is currently facing a severe challenge in population ageing. According to the latest data from the National Bureau of Statistics of China, the number of people aged 60 and older in China was 264 million, accounting for 18.7% of the total population on 1 November 2020 [1]. With the trend of population ageing, cognitive impairment and dementia among the elderly in China have increasingly become a public health problem. Cognitive impairment is a chronic disease that impairs an individual’s ability to remember, learn, concentrate, and make decisions in daily life [2]. Among people aged 60 and older in China, there are approximately 15.07 million people with dementia, including 9.83 million people with Alzheimer’s disease [3]. Cognitive impairment and dementia increase costs for governments, communities, families, and individuals, leading to a decline in economic productivity [4].

Many recent studies have demonstrated some risk factors for cognitive impairment and have found that age is the biggest risk factor for cognitive decline [5]. Moreover, the association between diet and cognitive impairment has been widely reported. Studies have shown that consuming fruits, vegetables, meat, and soybean-derived products could reduce the risk of cognitive impairment [6]. The higher the intake of fresh fish/shellfish, the lower the risk of cognitive impairment [7]. A national study in France showed that alcohol use disorders were major risk factors for various types of dementia, especially early-onset dementia [8]. In China, a study in Xi’an, Shaanxi Province, revealed a positive relationship between smoking and cognitive impairment [9], while another study in Zhejiang Province showed that passive smoking increased the risk of cognitive impairment in the elderly, especially in non-smokers [10]. Additionally, the association between modifiable physical characteristics and cognitive impairment has often been reported. Sleep problems could increase the risk of cognitive impairment [11], and poor oral health and activities of daily living are associated with it [12]. Being overweight is also related to a reduced risk of cognitive impairment in the Chinese elderly, while abdominal obesity is associated with an increased risk of cognitive impairment [13]. Chronic diseases are also associated with cognitive impairment. A U-shaped correlation between blood pressure and cognitive function has been found among the Chinese elderly [14]. Moreover, diabetes might be an independent risk factor for cognitive impairment, especially in the areas of language, visuospatial/executive functions, and attention [15].

Social support is an important concept related to loneliness and depression, which might be vital in combating depression in the elderly, but there is no widely accepted definition [16]. Shumaker and Brownell defined social support as an exchange of resources between at least two individuals perceived by the provider or the recipient to enhance the well-being of recipients [17]. Barker defined social support as a range of interpersonal relationships or connections that impact the individual’s functioning and generally included support provided by individuals and social institutions [16]. Lin described social support as perceived or actual instrumental or expressive provisions generally supplied by an individual’s partners, community, and social network [18]. Studies on the relationship between social support and other variables have mostly focused on a specific population, such as providing social support for pregnant women at each stage of the perinatal period to prevent depression [19], strengthening social support for patients with cancer to reduce suicidal thoughts [20], promoting support for patients with end-stage kidney disease to improve adherence to treatment [21], and enhancing support to improve the resilience and quality of life of patients with breast cancer [22].

In China, the effect of social support on mental health has been widely reported. Social support could help reduce depressive symptoms in patients with diabetes [23]. Perceived social support can reduce loneliness among adolescents [24]. Additionally, a study of Chinese seafarers showed that social support had a significant positive impact on health-related quality of life [25]. Improving the quantity and quality of social support might be an effective means of improving health literacy and reducing hospital admissions [26]. Further, social support networks play a significant role in promoting healthy ageing [27].

However, no prior studies specifically explored the association between social support and cognitive impairment in Chinese urban elderly, although a few studies have clarified this relationship among other Chinese population groups. Social support is negatively associated with the cognitive function of patients with peritoneal dialysis in China [28]. The Chinese Longitudinal Healthy Longevity survey showed that visits from children were consistently related to a low incidence of cognitive impairment in the elderly in China [29]. Less social support is an independent risk factor for subjective cognitive decline among the elderly in China [30]. Therefore, the purpose of this study was to understand the prevalence and risk factors of cognitive impairment among the urban elderly in Jinan, China.

## 2. Materials and Methods

### 2.1. Data Collection and the Research Participants

The data were collected in August 2020 in Jinan, Shandong Province, China. Shandong Province lies in the east of China. Jinan is the capital of Shandong Province, with a gross domestic product of CNY 1.01 trillion (approximately USD 158 billion) in 2020 [31]. As of 1 July 2020, Jinan has jurisdiction over 10 districts and 2 counties (132 sub-districts and 29 towns) [32]. By the end of 2019, the local resident population was 8.91 million—an increase of 0.78% over the previous year—while the registered population was 7.98 million, an increase of 1.46% [33]. The total urban population in Jinan City in 2019 was 5.96 million [34], which included 4.35 million urban residents and 1.6 million urban temporary residents; this study included Jinan City urban elderly who were older than 60 years of age.

The method of multi-stage cluster random sampling was used to recruit participants. In the first stage, two districts were chosen among 10 districts as the primary sampling units, comprehensively considering economic development and geographic location. In the second stage, two sub-districts were selected from each primary sampling unit as the secondary sampling units, which means that one sub-district was chosen from each of the previously selected districts. In the third stage, two communities were selected from the secondary sampling units, which means that one community was chosen from each of the previously selected sub-districts. All the urban elderly in these two communities constituted the total sample of this study.

The inclusion criteria of the respondents were adults aged ≥ 60 years living in Jinan City who were informed about the content of this study and could communicate. To obtain complete and accurate data, we excluded participants who could not communicate normally and were unwilling to cooperate with the interview owing to their physical condition or other reasons.

Thirty-two university students became the investigators after receiving training concerning the study background, questionnaire contents, and social survey techniques. Of the 32 investigators, 11 were from Shandong University, 13 from Jinan University, 2 from Dongying Vocational Institute, and 7 from Weifang Medical University. Twenty-minute face-to-face interviews were conducted between the investigators and the participants to collect the data. A total of 522 elderly people were initially chosen and interviewed; however, 10 were excluded from the sample owing to obvious logical errors or incomplete questionnaires. The data from 512 elderly individuals were thus analysed.

### 2.2. Socio-Demographic Characteristics

The socio-demographic characteristics included sex (male/female), age (years), height (centimetres), weight (kilogrammes), religion (yes/no), marital status (unmarried, divorced, or widowed/married), employment status (unemployed, retired, employed), educational level (university and above, high school, primary school, uneducated), occupation/pre-retirement occupation (mental-related work, physical-related work, mental and physical work), source of living expenses (basic living allowances, relies on others, relies on self) and monthly income (renminbi).

Body mass index (BMI) was calculated as weight divided by height squared (kg/m^2^). A BMI < 18.5 kg/m^2^ was considered underweight, between 18.5 and 23.9 kg/m^2^ was considered normal, between 24 and 27.9 kg/m^2^ was considered overweight, and ≥ 28 kg/m^2^ was considered obese [35].

### 2.3. Social Support

The Social Support Rating Scale (SSRS) was used to evaluate the level of social support. The SSRS was developed by Xiao Shuiyuan in 1994, who specially designed it for Chinese individuals. It contains 10 questions and the following 3 dimensions: subjective support, objective support, and support utilisation [36]. Total scores range from 12 to 66. Higher scores indicate higher levels of social support. Scores between 12 and 22 indicate low, between 23 and 44 indicate moderate, and between 45 and 66 indicate high social support.

### 2.4. Cognitive Function

The Mini-Mental State Examination scale was used to assess cognitive function. Total scores range from 0 to 30; higher scores indicate better cognitive function. The critical value of cognitive impairment depends on educational level. Cognitive impairment was defined as a score of 17 or lower for illiterate participants, 20 or lower for participants with primary school education, and 24 or lower for those with at least a junior high school education [37].

### 2.5. Statistical Analysis

All statistical analyses were performed using SPSS 24.0 (IBM Corp., Armonk, NY, USA). The reported confidence intervals (CIs) were calculated at the 95% level, and *p*-values < 0.05 were deemed significant. First, we used frequency and percentage to describe participants’ socio-demographic and social support characteristics. Chi-square tests were used to compare the differences in socio-demographic and social support characteristics between the elderly with and without cognitive impairment. Lastly, univariate and multivariate logistic regression analyses were used to explore the determinants of cognitive impairment in the elderly. Three binary logistic regression models were used to identify the differences in cognitive impairment among the elderly with different degrees of social support: Model 1, the socio-demographic factors; Model 2, the univariate model; and Model 3, all factors in Models 1 and 2.

## 3. Results

### 3.1. Participants’ Basic and Social Support Characteristics

Table 1 shows participants’ socio-demographic and social support characteristics. The study included 512 participants, of which 35.55% were men and 64.45% were women. Overall, 24.4% of the participants had cognitive impairment. The average age of all participants was 69.66 ± 8.91 years. The BMI results showed that almost half of the participants were overweight (45.12%). Most participants had no religion (96.09%), were married (76.17%), retired (75.39%), and had a high-school education (61.91%). More than half of the participants were engaged in mental-related work before retirement (51.95%), and 81.05% relied on themselves to pay for living expenses. Most had a moderate level of social support (68.75%). There were significant differences in cognitive impairment depending on age (*p* < 0.001), religion (*p* = 0.022), marital status (*p* = 0.001), employment status (*p* = 0.018), educational level (*p* < 0.001), occupation/pre-retirement occupation (*p* < 0.001), source of living expenses (*p* = 0.041), and total SSRS score (*p* < 0.001).

### 3.2. Association between Social Support and Cognitive Impairment

Table 2 shows the relationship between socio-demographic factors, social support, and cognitive impairment. In Model 1, the results showed that people aged ≥ 73 years were more likely to experience cognitive impairment than those aged ≤ 64 years (Odds Ratio (OR) = 2.530, 95% Confidence Interval (CI) = 1.345–4.759, *p* = 0.004). The risk of cognitive impairment among participants with an education level of primary school was higher than that of those with a level of university and above (OR = 4.381, 95% CI = 1.402–13.691, *p* = 0.011). Regarding occupation/pre-retirement occupation, participants who had been engaged in physical-related work had a higher risk of cognitive impairment than those who had been engaged in mental-related work (OR = 2.311, 95% CI = 1.333–4.007, *p* = 0.003).

Model 2 illustrated that there were significant differences between all levels of social support and cognitive impairment (OR = 31.667, 95% CI = 3.524–284.596, *p* = 0.002 for moderate social support; OR = 2.478, 95% CI = 1.480–4.150, *p* = 0.001 for low social support).

In Model 3, the statistical association between the corresponding variables and cognitive impairment was similar to that in Models 1 and 2. Concerning age, the OR value of ≥ 73 years was 2.410 (95% CI = 1.275–4.553, *p* = 0.007). Regarding educational level, the OR value of primary school level was 4.313 (95% CI = 1.371–13.564, *p* = 0.012). As for occupation/pre-retirement occupation, the OR value of physical-related work was 2.216 (95% CI = 1.271–3.863, *p* = 0.005). Regarding the total SSRS score, the OR value for moderate social support was 12.177 (95% CI = 1.244–119.216, *p* = 0.032), while that for low social support was 1.854 (95% CI = 1.061–3.240, *p* = 0.030).

## 4. Discussion

### 4.1. Socio-Demographic Factors and Cognitive Impairment

At present, cognitive impairment and dementia cannot be cured, and the available treatments are only moderately effective. Therefore, it is critical to identify the potential risk factors of cognitive impairment to prevent or reduce it. This study collected data on the cognitive function, social support, and socio-demographic characteristics of the participants to explore the factors related to cognitive impairment.

The prevalence of cognitive impairment in the present study was 24.4%. This was less than a study conducted with urban and rural elderly greater than 65 years (average age = 87 years old) in China, whose prevalence rate was 38.5% [14]. In another study that focused on rural elderly aged greater than 65 years (average age = 73 years old), the prevalence of cognitive impairment was 42.9% [38], which was also higher than in the current study. The lower prevalence rate of cognitive impairment in this study might be owing to the age-inclusion criteria of the elderly (aged ≥ 60 years) and the mean age of the sample (69.66 years), which were less than both studies above. Furthermore, all the current participants were elderly adults living in cities, while those living in rural areas are more likely to experience cognitive impairment [39].

The results suggested that people aged ≥ 73 years were more likely to experience cognitive impairment than those aged ≤ 64 years. These results are consistent with prior results [40,41]. Age is a major risk factor for cognitive impairment and a convenient proxy for an array of cellular and molecular mechanisms that underlie age-related cognitive impairment [42]. These include decreased levels of synaptic proteins, such as neuronal pentraxin II [43], as well as oxidative stress, increased reactive oxygen species production, decreased brain lysosomal function [44] and mitochondrial dysfunction [45].

Lack of formal school education is related to a high incidence of cognitive impairment [38]. In a study of changes in cognitive impairment and its risk factors among the elderly in China from 2005 to 2014, the results showed that people with low education levels were at a higher risk of cognitive impairment than their counterparts [46]. A retrospective cohort study in Colombia also supported that educational level might be a protective factor to prevent the onset of cognitive impairment [47]. A study from South Korea found that education and occupation could reduce cognitive impairment caused by stroke and promote rapid cognitive recovery in the early stages of stroke [48]. The results of this study coincide with these prior findings.

A meta-analysis of occupational factors associated with cognitive impairment or dementia showed that those who performed mental work were less likely to experience mild cognitive impairment than those who performed other types of work. Moreover, higher work complexity could reduce the risk of dementia [49], which is consistent with the present results. Regarding the specific types of occupations, former blue-collar elderly workers or homemakers were more likely to experience cognitive deficits in Italy than their counterparts [50]. Additionally, a study in Puerto Rico suggested that an increased complexity of work might reduce the risk of cognitive impairment in the elderly [51].

### 4.2. Social Support and Cognitive Impairment

A significant association between social support and cognitive impairment was observed in this study. Some previous studies found that a higher level of social support indicates a lower risk of cognitive impairment [30,52], while other studies came to the opposite conclusion—that a higher level of social support would generally indicate a higher risk of cognitive impairment [53,54]. The current results demonstrated that participants with a moderate level of social support were most likely to experience cognitive impairment, followed by those with low social support. However, only 6 elderly people in this study had a low level of social support, so it is open to debate whether the risk of cognitive impairment in elderly people with low social support is lower than that for those with moderate social support. Social activities might include a cognitive stimulus that promotes cognitive outcomes by establishing cognitive reserves, and these reserves optimise cognitive performance by recruiting alternate brain networks and cognitive strategies to compensate for cognitive difficulties associated with pathology [55]. Social support could affect cognitive outcomes through a buffering effect on stress [55].

### 4.3. Limitations

This study had some limitations. First, this study was cross-sectional, and there is no way to confirm causality; a follow-up study is needed in the future. Considering the possibility that there is a sort of bidirectional relationship between social support and cognitive impairment, a follow-up study addressing causality might also help to distinguish between the protective effect of social support on the one hand and intensified social support as a consequence of cognitive impairment on the other. Second, the data were self-reported, which made recall and reporting bias inevitable. Third, the participants in this study were all from Jinan, Shandong Province; thus, the conclusions cannot be extended to other parts of China. Finally, there might be other, overlooked confounding factors in this study, which could be included in future studies.

### 4.4. Implications

No prior studies explored the relationship between social support and cognitive impairment in Chinese urban elderly. This study found that social support and the related socio-demographic factors were significantly associated with cognitive impairment in older adults in urban communities, which provide useful information for cognitive impairment intervention. First, the government should pay more attention to health education on the cognition of the elderly, given that elderly cognition is often ignored due to a lack of awareness. The government should conduct a related education programme to spread awareness in the community and enable the elderly with cognitive impairments to see a physician promptly. Second, family members of the elderly should strengthen their knowledge about cognitive impairment, pay close attention to the psychological needs of their elderly loved ones, and ensure they obtain the necessary social support. Finally, healthy elderly adults should receive appropriate cognitive training and maintain active social communication to prevent or reduce the occurrence of cognitive impairment.

## 5. Conclusions

The prevalence of cognitive impairment was 24.4% among urban elderly in Jinan, China. Age, educational level, occupation or pre-retirement occupation, and social support were significantly associated with participants’ cognitive impairment. Those aged ≥ 73 years, with an education level of primary school, who performed physical-related work, and had only a moderate level of social support were more likely to experience cognitive impairment than those aged ≤ 64 years, with an education level of university and above, who performed mental-related work, and had a high level of social support. These results provide evidence-based information that can inform the government and the public on how to prevent or decrease cognitive impairment among the elderly in Jinan, China.

## Figures and Tables

**Table 1:** Socio-demographic characteristics and social support of the elderly in Jinan, China.

Variable	*n* (%)	Cognitive Impairment		χ^2^	*p*
		No	Yes		
		*n* (%)	*n* (%)		
**Total**	512 (100.00)	387 (75.60)	125 (24.40)		
**Sex**				0.113	0.736
Male	182 (35.55)	136 (35.14)	46 (36.80)		
Female	330 (64.45)	251 (64.86)	79 (63.20)		
**Age**				21.245	0.001
≤64	166 (32.42)	142 (36.69)	24 (19.20)		
65–72	169 (33.01)	131 (33.85)	38 (30.40)		
≥73	177 (34.57)	114 (29.46)	63 (50.40)		
**BMI**				1.233	0.745
≤18.4	15 (2.93)	12 (3.10)	3 (2.40)		
18.5–23.9	190 (37.11)	139 (35.92)	51 (40.80)		
24.0–27.9	231 (45.12)	179 (46.25)	52 (41.60)		
≥28.0	76 (14.84)	57 (14.73)	19 (15.20)		
**Religion**				6.694	0.022
Yes	20 (3.91)	18 (4.65)	2 (1.60)		
No	492 (96.09)	369 (95.35)	123 (98.40)		
**Marital status**				11.783	0.001
Unmarried, divorced or widowed	122 (23.83)	78 (20.16)	44 (35.20)		
Married	390 (76.17)	309 (79.84)	81 (64.80)		
**Employment status**				7.999	0.018
Unemployed	102 (19.92)	67 (17.31)	35 (28.00)		
Retired	386 (75.39)	299 (77.26)	87 (69.60)		
Employed	24 (4.69)	21 (5.43)	3 (2.40)		
**Educational level**				28.734	0.001
University and above	50 (9.77)	46 (11.89)	4 (3.20)		
High school	317 (61.91)	253 (65.37)	64 (51.20)		
Primary school	101 (19.73)	59 (15.25)	42 (33.60)		
Uneducated	44 (8.59)	29 (7.49)	15 (12.00)		
**Occupation/Pre-retirement occupation**				16.262	0.001
Mental-related work	266 (51.95)	220 (56.85)	46 (36.80)		
Physical-related work	187 (36.52)	124 (32.04)	63 (50.40)		
Mental and physical work	59 (11.52)	43 (11.11)	16 (12.80)		
**Source of living expenses**				6.369	0.041
Basic living allowances	8 (1.56)	6 (1.55)	2 (1.60)		
Relies on others	89 (17.38)	58 (14.99)	31 (24.80)		
Relies on self	415 (81.05)	323 (83.46)	92 (73.60)		
**Monthly income**				5.097	0.278
Q1 (poorest)	102 (19.92)	70 (18.09)	32 (25.60)		
Q2	72 (14.06)	59 (15.25)	13 (10.40)		
Q3	133 (25.98)	102 (26.36)	31 (24.80)		
Q4	102 (19.92)	75 (19.38)	27 (21.60)		
Q5 (richest)	103 (20.12)	81 (20.93)	22 (17.60)		
**Total SSRS score**				22.326	0.001
High (≥45)	154 (30.08)	133 (34.37)	21 (16.80)		
Moderate (23–44)	352 (68.75)	253 (65.37)	99 (79.20)		
Low (≤22)	6 (1.17)	1 (0.26)	5 (4.00)		

BMI = body mass index; Q1 = Poorest, Q2 = Relatively poorer, Q3 = Neither poor nor rich, Q4 = Relatively rich, Q5 = Richest; SSRS = Social Support Rating Scale.

**Table 2:** Logistic regression analysis on the relationship between socio-demographic factors, social support and cognitive impairment.

Variables	Model 1			Model 2			Model 3		
	OR	95% CI	*p*	OR	95% CI	*p*	OR	95% CI	*p*
**Age**									
≤64	1.000						1.000		
65–72	1.435	0.782–2.633	0.243				1.384	0.750–2.552	0.299
≥73	2.530	1.345–4.759	0.004				2.410	1.275–4.553	0.007
**Religion**									
Yes	1.000						1.000		
No	1.931	0.424–8.803	0.395				2.002	0.439–9.139	0.370
**Marital status**									
Unmarried, divorced or widowed	1.000						1.000		
Married	0.663	0.401–1.099	0.111				0.770	0.458–1.296	0.326
**Employment status**									
Unemployed	1.000						1.000		
Retired	0.508	0.120–2.143	0.356				0.617	0.143–2.661	0.517
Employed	0.990	0.432–2.265	0.980				1.005	0.436–2.321	0.990
**Educational level**									
University and above	1.000						1.000		
High school	2.618	0.719–9.536	0.145				2.390	0.643–8.879	0.193
Primary school	4.381	1.402–13.691	0.011				4.313	1.371–13.564	0.012
Uneducated	2.577	0.871–7.621	0.087				2.720	0.915–8.085	0.072
**Occupation/Pre-retirement** **occupation**									
Mental-related work	1.000						1.000		
Physical-related work	2.311	1.333–4.007	0.003				2.216	1.271–3.863	0.005
Mental and physical work	1.263	0.622–2.566	0.519				1.593	0.771–3.293	0.209
**Source of living expenses**									
Basic living allowances	1.000						1.000		
Relies on others	1.321	0.235–7.421	0.752				1.090	0.190–6.253	0.923
Relies on self	1.491	0.265–8.378	0.650				1.154	0.200–6.669	0.873
**Total SSRS score**									
High (≥45)				1.000			1.000		
Moderate (23–44)				31.667	3.524–284.596	0.002	12.177	1.244–119.216	0.032
Low (≤22)				2.478	1.480–4.150	0.001	1.854	1.061–3.240	0.030

Notes: OR = odds ratio, CI = confidence interval, SSRS = Social Support Rating Scale.

## Data Availability

Additional datasets analysed during the current study are available from the corresponding author on reasonable request.
